# Behçet's Disease and Autoimmune Atrophic Gastritis: An Incidental Finding

**DOI:** 10.1155/carm/5813761

**Published:** 2025-06-13

**Authors:** Philippe Attieh, Antonio Al Hazzouri, Rose-Mary Daou, Sara El Haddad, Karam Karam, Elias Fiani

**Affiliations:** ^1^Department of Internal Medicine, University of Balamand, Beirut, Lebanon; ^2^Department of Gastroenterology and Hepatology, University of Balamand, Beirut, Lebanon

**Keywords:** aphthous ulcer, autoimmune atrophic gastritis, Behçet's disease

## Abstract

Behçet's disease (BD) is a systemic inflammatory condition causing oral ulcers, genital sores, eye inflammation, and skin lesions. Autoinflammatory and autoimmune disorders are chronic immune system activation leading to tissue inflammation. Current evidence suggests that BD is at the intersection of autoimmune and autoinflammatory syndromes, with some findings suggesting an autoinflammatory nature. Oral aphthous ulcers are the commonest initial manifestation of the disease. Gastric manifestations in BD are infrequent. The usually seen finding in the stomach is either ulcers or gastritis, presenting as epigastric pain. BD has been linked with several autoimmune diseases; however, it has not yet been seen with autoimmune atrophic gastritis. We present a case of a 62-years-old male patient presenting for oral aphthous ulcers with vague abdominal pain, epigastric discomfort, and postprandial nausea. The patient was positive for HLA-B5 alleles, leading to a diagnosis of BD. Gastroscopy and colonoscopy were done to investigate a probable etiology for this patient's epigastric discomfort and abdominal pain. Gastroscopy showed multiple erosions at the level of the fundus and atrophic folds at the level of the body of the stomach, but no important findings were seen on colonoscopy. Furthermore, a gastric biopsy was done and confirmed the presence of autoimmune atrophic gastritis at the level of the fundus and antrum of the stomach which is atypical in BD that is commonly associated with aphthous ulcerations at the level of the terminal ileum. To our knowledge, this is the first case reported, which should prompt for further investigation behind the mechanism linking these two diseases.

## 1. Introduction

Behçet's disease (BD) is a chronic, relapsing, and remitting vasculitis capable of affecting multiple organ systems, including mucocutaneous, ocular, gastrointestinal (GI), urologic, and others. While the ileocecal region is the most commonly affected site in BD, involvement of the entire GI tract, including the upper GI (UGI) tract, has also been documented. UGI inflammation in BD can result in severe complications such as bleeding, perforation, or fistula formation, emphasizing the importance of recognizing UGI involvement for effective disease management. The middle and lower parts of the esophagus are the most frequently affected UGI sites, often presenting with various types of ulcers, including single round or multiple oval-shaped lesions. Gastroduodenal involvement, although less common, has been described, with characteristic findings such as punched-out ulcers in the gastric antrum and multiple small ulcers in the descending duodenum [1]. This report presents the case of a 62-year-old male who presented with oral aphthous ulcers, epigastric discomfort, and postprandial nausea. The patient, who tested positive for HLA-B5, underwent a colonoscopy that revealed no abnormalities, including in the terminal ileum. However, gastroscopy findings showed atrophic gastric folds and an absence of fundal gastric patterns, features characteristic of autoimmune atrophic gastritis (AAG), which was subsequently confirmed through UGI endoscopy and biopsy. This case underscores a potential link between BD and AAG, as BD is an autoinflammatory process, which can suggest that the mechanism linking both diseases is based on autoimmune cells. Furthermore, this case highlights the importance of considering upper GI endoscopy in patients with BD.

## 2. Case Presentation

**Figure 1:**
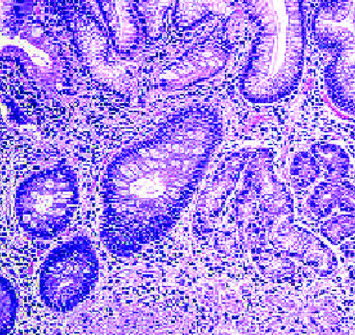
Histology showing elongated crypts and an enlarged chorion with abundant, mononuclear, inflammatory infiltrate, and rich in polynuclear eosinophils.

**Figure 2:**
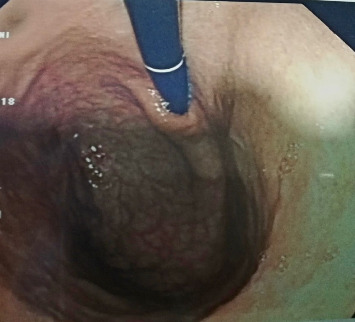
Autoimmune atrophic gastritis at the level of the stomach antrum and fundus.

A 62-year-old male patient, previously healthy with no past surgical history, nonsmoker with no known food and drug allergies, and not on chronic medications, presented to the clinic for oral aphthous ulcers accompanied by vague abdominal pain, epigastric discomfort, and postprandial nausea. Previously, the patient did an immunoassay for HLA subtypes which were found to be positive for HLA-B5 alleles consistent with BD. Colonoscopy was done and results were totally normal. Gastroscopy was then done and showed multiple erosions at the level of the fundus and atrophic folds at the level of the body of the stomach, highly suggestive of BD. Biopsies taken showed a reduced thickness of antral and fundal mucosa at the level of the stomach. The fundal mucosa presents elongated crypts and an enlarged chorion with abundant, mononuclear, inflammatory infiltrate, and is rich in polynuclear eosinophils. Focal exocytosis of lymphocytes in the epithelium is observed without producing true lymphoepithelial lesions. Numerous enterocytic goblet cells are present, indicating complete intestinal metaplasia (Figure 1). There was no evidence of Helicobacter pylori. These upper endoscopy findings were suggestive of AAG (Figure 2).

## 3. Discussion

BD is a systemic inflammatory condition marked by recurring oral ulcers, genital sores, eye inflammation, and skin lesions. Although less common, involvement of large blood vessels, the central nervous system, the GI tract, and thrombotic events can be severe and life-threatening. Among the complications, ocular involvement significantly impacts patients' quality of life, with 50%–90% of individuals experiencing intraocular inflammation. Autoinflammatory and autoimmune disorders (AID) are both considered aberrant chronic activation of the immune system eventually leading to tissue inflammation. However, the pathogenesis of autoinflammatory disease is linked to innate immunity while AID are mediated through the adaptive immunity. Based on current evidence, BD does not match any one of these two mechanisms. In fact, BD is at the crossroad between autoimmune and autoinflammatory syndromes with some findings suggestive that BD has an autoinflammatory nature. These include a high neutrophils activity and elevated levels of interleukin-1β in both BD and autoinflammatory diseases, recurrent episodes of remission and exacerbation, absence of specific autoantibody attributed to the pathogenesis of BD, in opposite to other AID, and the relationships between BD and some autoinflammatory diseases like FMF with both diseases having a particular variant of the MEFV gene [2].

Several studies have documented the coexistence of BD with autoimmune inflammatory diseases. For example, BD has been associated with conditions such as systemic lupus erythematosus (SLE), rheumatoid arthritis (RA), autoimmune thyroiditis, and inflammatory bowel disease (IBD), particularly Crohn's disease, which shares GI features with AAG [3, 4]. These findings suggest a broader immunological dysregulation that may predispose BD patients to additional autoimmune pathologies.

Oral aphthous ulcers are the commonest initial manifestation of the disease (70%) and can recur in 90%–100% of cases. Gastric manifestations in BD are infrequent and usually seen as either ulcers or gastritis. A study from Taiwan, which included 28 BD patients, has shown a 43% prevalence rate of gastric/duodenal ulceration thought to be induced by vasculitis and responding well to corticosteroids and immunosuppressant drugs, rather than to conventional H2-blockers. Rare gastric complications include pyloric stenosis and Dieulafoy's ulcer. The intestine and esophagus can also be involved in BD with the formation of ulcers [5]. The International Study Group for BD established diagnostic criteria requiring patients to have recurrent aphthous stomatitis (RAS) along with at least two of the following: recurrent genital ulcers, eye involvement, skin lesions, or a positive pathergy test [6].

Common Variable Immunodeficiency (CVID) patients have been found to be more susceptible to AID and AAG, requiring regular endoscopic surveillance for these individuals. Overall, AID occurs in approximately 25%–30% of patients with CVID. This was explained by a possible mechanism that CVID patients cannot completely eliminate microbes due to immunoglobulin deficiencies, favoring the deposition of immune complexes, leading to the activation of autoreactive T cells. Due to abnormal regulatory mechanisms of the immune system, there is reduction in the apoptosis of autoreactive T cells and dysregulate cytokine production [7].

A study analyzing 870 pediatric and adult patients with CVID from the USIDNET registry revealed that Sjögren's disease, SLE, and Behçet's syndrome were associated with CVID. The GI system is often involved in CVID, making the diagnosis of GI AID, such as celiac disease and AAG, particularly challenging. Autoimmune gastritis, characterized by the progressive atrophy of the gastric corpus and fundus mucosa, leads to malabsorption of vitamin B12 and iron. [7]. Another study mainly assessed the frequencies of serum gastric parietal cell antibody (GPCA), thyroglobulin antibody (TGA), and thyroid microsomal antibody (TMA) positivities in 30 atrophic glossitis (AG)-positive RAS/BD (AGþRAS/BD) and 33 AG-negative RAS/BD (AG־RAS/BD) patients. AG-positive BD patients (AG + RAS/BD) had significantly higher rates of GPCA, TGA, and TMA compared to healthy controls. The study suggests that the presence of AG in BD patients is associated with higher frequencies of autoantibodies, indicating a potential link between AG and autoimmune activity in BD [6].

A second study explores the prevalence of GPCA, TGA, and TMA in patients with BD, compared to patients with RAS and healthy controls. BD patients showed higher positivity for GPCA, TGA, and TMA compared to healthy controls. However, these rates were not significantly higher than those observed in RAS patients. These findings imply the importance of monitoring BD patients for potential autoimmune comorbidities, in order to early identify and manage GPCA-positive or TGA/TMA-positive patients to prevent complications like pernicious anemia or thyroid dysfunction [8]. Genetic susceptibility, particularly HLA-B51 allele, plays a pivotal role in BD, alongside variants in IL-10, which reduce anti-inflammatory cytokine production, and IL-23R, which promotes Th17-mediated inflammation. Bacterial (*Streptococcus sanguis*, *Helicobacter pylori*) and viral infections activate the innate and adaptive immune systems through molecular mimicry, with microbial antigens resembling host proteins like heat shock proteins, stimulating autoimmune responses [9]. Once the innate immune system is activated, neutrophils are hyperactivated, showing increased chemotaxis and reactive oxygen species production, which damages vascular endothelial cells. Natural Killer cells, particularly the CD56dim subset, contribute to inflammation by producing interferon-gamma (IFN-γ). Furthermore, γδ T cells are activated by microbial antigens and produce pro-inflammatory cytokines such as TNF-α and IL-17, which perpetuate inflammation and recruit additional immune cells [9]. In the adaptive immune system, there is an imbalance between proinflammatory Th1 and Th17 cells, and anti-inflammatory regulatory T cells (Tregs). Th1 and Th17 cells dominate in BD, producing cytokines like IFN-γ, TNF-α, and IL-17A, which contribute to chronic inflammation and tissue damage [9]. Cytokines play a critical role in BD's pathogenesis. Elevated levels of IL-1β, IL-6, TNF-α, and IL-17 are central to sustaining inflammation, while anti-inflammatory cytokines like IL-10 and IL-37 are insufficiently expressed [9].

In our case, our patient had BD with atypical findings. His colonoscopy was unexpectedly normal, with an upper GI endoscopy and biopsy consistent with AAG. He had no other clinical conditions like CVID or other AID. Although AAG is not a particularly rare condition, occurring in approximately 1% of the general population, its occurrence in a patient with BD raises the question of whether this association is causal or merely coincidental. Given the rarity of coexisting organ-specific autoimmune diseases in BD, the possibility of incidental coexistence cannot be excluded. However, current evidence increasingly supports the idea that BD exists at the crossroads between autoinflammatory and autoimmune conditions. In this context, it is plausible that certain patients with BD may be predisposed to develop organ-specific autoimmune manifestations, including AAG, particularly in the absence of other confounding immunodeficiencies such as CVID. While we do not claim a definitive causal relationship, this case draws attention to a potentially under-recognized clinical association. Therefore, we propose that such findings merit further investigation to determine whether a shared immunological mechanism exists or whether this combination is an infrequent overlap without pathological significance. To our knowledge, this case is the first of its kind; in addition, considering an upper GI endoscopy for patients with BD should be brought up.

## 4. Conclusion

This case highlights a rare manifestation in patients with BD which is the presence of AAG at the level of the fundus and antrum of the stomach confirmed by UGI endoscopy which is atypical in BD, knowing that the latter is characterized by the presence of aphthous ulcers in terminal ileum that were not seen on colonoscopy in our case. This raises the importance on performing both colonoscopy and UGI endoscopy when encountering a patient with BD and to look for the underlying immune mechanism that links these two diseases.

## Data Availability

The data that support the findings of this study are available from the corresponding author upon reasonable request.
